# Dataset showing the relationship between terpenes, antioxidants and polyphenols in protecting ester and ether linked glycerophospholipids of grilled beef and moose meat marinated with unfiltered beer from oxidation

**DOI:** 10.1016/j.dib.2020.106324

**Published:** 2020-09-22

**Authors:** Charles F. Manful, Natalia P. Vidal, Thu H. Pham, Muhammad Nadeem, Evan Wheeler, Raymond H. Thomas

**Affiliations:** School of Science and the Environment/Boreal Ecosystem Research Initiative, Grenfell Campus, Memorial University of Newfoundland, Corner Brook, NL A2H 5G4, Canada

**Keywords:** Marinades, Lipid oxidation, Antioxidants, Polyphenols, Grilled food quality, Oxygenated terpenes

## Abstract

This article presents the associated data set in the research article entitled “Assessing beer-based marinades effects on ether and ester linked phosphatidylcholines and phosphatidylethanolamines in grilled beef and moose meat” published in *Meat Science* [1], demonstrating the use of unfiltered beer-based marinades in improving the nutritional quality of grilled ruminant meat by suppressing the degradation of health-promoting ester and ether-linked PC and PE the most predominant glycerophospholipids (GPL) in meat. High throughput lipidomics analysis was conducted using high-resolution accurate mass tandem mass spectrometry (UHPLC—HRAMS/MS-MS) to profile the meat lipids following marination and grilling. The marinades were composed of a combination of unfiltered beers, fruits, herbs and spices. The data presented show the retention levels of ether as well as ester linked PC and PE molecular species; Pearson's correlations for the associations between antioxidants, phenolics, volatile oxygenated terpenes, oxidation status and preserved phospholipid species in the marinated grilled meats. There are many studies demonstrating cooking effects on fatty acid composition of meat phospholipids in the literature. However, information on how marination and grilling affects intact ether and ester linked PC and PE composition in grilled ruminant meats is limited. As such, this dataset provides useful information on the preservation of ruminant meat ester and ether-linked glycerophospholipid composition following marination with unfiltered beer-based marinades and meat preparation via grilling. Specifically, this data demonstrate the preservation of ether and ester linked PC and PE enriched with essential ω3 and ω6 fatty acids from degradation during grilling. For additional insights see [1] DOI: 10.1016/j.meatsci.2020.108271.

## Specifications Table

**Table:** 

Subject	Agricultural and Biological Sciences
Specific subject area	Food Science
Type of data	Figures Tables
How data were acquired	Data were acquired by the extraction of meat lipids from marinated and unmarinated grilled meats and subsequent analysis of intact PC and PE lipid species by UHPLC—HRAMS/MS-MS. Raw lipid data was processed using LipidSearch 4.2 (Mitsui Knowledge Industry, Tokyo, Japan) and Xcalibur 4.0 (Thermo Fisher Scientific, Ontario, Canada) softwares to detect PC and PE lipid species. Detected PE and PE lipid species were identified and semi-quantified using the peak areas based on internal standard normalization, and concentrations were expressed as nmol%. Total ether and ester linked PC and PE content (mg/100 g FW meat) of grilled meat samples was also calculated based on standard curves generated from authenticated PC and PE standards contained in SPLASH® Lipidomix® Mass Spec Standard (Avanti Polar Lipids, Alabama, USA).
Data format	Raw Analyzed
Parameters for data collection	Lipids were extracted from marinated and unmarinated grilled beef and moose meat by Folch method [2,3]. Extracted lipids were resolved using an Accucore C30 RP column (150 × 2 mm I.D., particle size: 2.6 µm, pore diameter: 150 Å; Thermo Fisher Scientific, ON, Canada) installed on a Dionex Ultimate 3000 ultra-high performance liquid chromatography (UHPLC) system coupled to a Q-Exactive Orbitrap high resolution mass spectrometer (Thermo Fisher Scientific, ON, Canada) [4].
Description of data collection	Three replicates (*n* = 3) were employed per experimental treatment. One-way analysis of variance (ANOVA) was used to determine if there were significant differences between PC and PE retained in the marinated grilled meats compared to the control. Where treatment effects were significant, the means were compared with Fisher's Least Significant Difference (LSD), at *α* = 0.05. Pearson's correlation coefficients were used to determine associations between preserved PC and PE species, antioxidant activities, phenolic contents, oxygenated terpenes, and oxidation status of marinated grilled meat samples. All statistical analysis was performed using XLSTAT Premium Version (Addinsoft, NY, USA).
Data source location	Memorial University of Newfoundland, Corner Brook, Newfoundland, Canada
Data accessibility	With the article
Related research article	C.F. Manful, T.H. Pham, M. Nadeem, E. Wheeler, K.J. Warren, N.P. Vidal, and R.H. Thomas, Assessing unfiltered beer-based marinades effects on ether and ester linked phosphatidylcholines and phosphatidylethanolamines in grilled beef and moose meat*.* Meat Science, 2020: p. 108,271. doi:10.1016/j.meatsci.2020.108271S.

## Value of the Data

• The functional and nutritional properties of dietary GPL depend on their structure and fatty acid compositions. The levels of GPL molecular species in unfiltered beer-based marinated grilled beef and moose meat provides useful information on effects of dietary antioxidants, polyphenols and volatile oxygenated monoterpenes on the retention, composition and nutritional quality of grilled ruminant meats.
• The correlations between GPL molecular species, antioxidants, polyphenols, volatile oxygenated monoterpenes and the oxidation status of grilled meats provides a promising strategy to reduce oxidative degradation of meat GPL. This approach could be very effective in producing grilled meat with superior dietary lipids, antioxidants, and nutritional quality.
• The data will help to better understand the potential benefits of unfiltered beer-based marinades in preserving the nutritional quality and health promoting dietary lipids in grilled meat.

## 1 Data Description

**Fig. 1:**
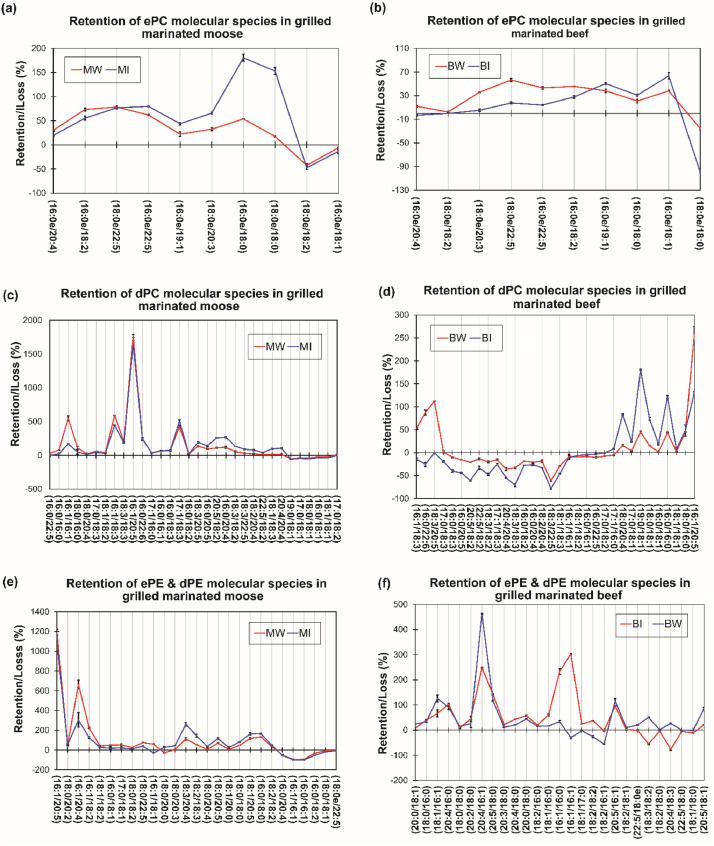
Parallel plots showing marination effects on ether and ester PC and PE linked molecular species in grilled beef and moose meats. Values in plots represent means ± standard errors. Plots showing percentage changes (%) in ePC **(a-b)** dPC **(c-d)** ePE and dPE **(e-f)** molecular species distribution in moose and beef respectively. [BC, MC] = unmarinated grilled beef and moose; [BI, MI] = Indian session ale-based marinated grilled beef and moose; [BW, MW] = Wheat ale-based marinated grilled beef and moose. PC = Phosphatidylcholine; PE = Phosphatidylethanolamine; *e* = Ether; *d* = Diacyl.

The data set contains retention levels of intact ether and ester linked PC and PE species detected in unfiltered beer-based marinated grilled meat samples, as well as the Pearson's correlations tables showing association of antioxidants, polyphenols, volatile oxygenated terpene compounds present in the marinades and the preserved ether and ester linked PC and PE species in the grilled meats. The relationship between preservation of PC and PE species and oxidation levels of marinated grilled meats is also included to show the reduction in oxidation levels with marination using the unfiltered beer-based marinades. Fig. 1 shows retention levels of ether PC (ePC), diacyl PC (dPC), ether PE (ePE), and diacyl PE (dPE) molecular species in marinated grilled beef and moose meats while Figure 3 shows retention levels of ester linked lyso PC (LPC), lyso PE (LPE) as well as ether linked lyso PC(eLPC), and lyso PE (eLPE) molecular species in marinated grilled beef and moose meats. Table 1 shows Pearson's correlation coefficients for relationships between antioxidant activities, phenolic contents, volatile oxygenated terpenes, oxidation status, and the preserved ePC molecular species in grilled moose and beef meats. Table 2 and Table 3 show Pearson's correlation coefficients for relationships between antioxidant activities, phenolic contents, volatile oxygenated terpenes, oxidation status, and the preserved dPC molecular species in grilled moose and beef respectively. In similar fashion, Table 4 and Table 5 show Pearson's correlation coefficients for relationships between antioxidant activities, phenolic contents, volatile oxygenated terpenes, oxidation status, and the preserved ePE and dPE molecular species in grilled moose and beef respectively. The raw data file is included as supplementary material in this data in brief article.

**Fig. 2:**
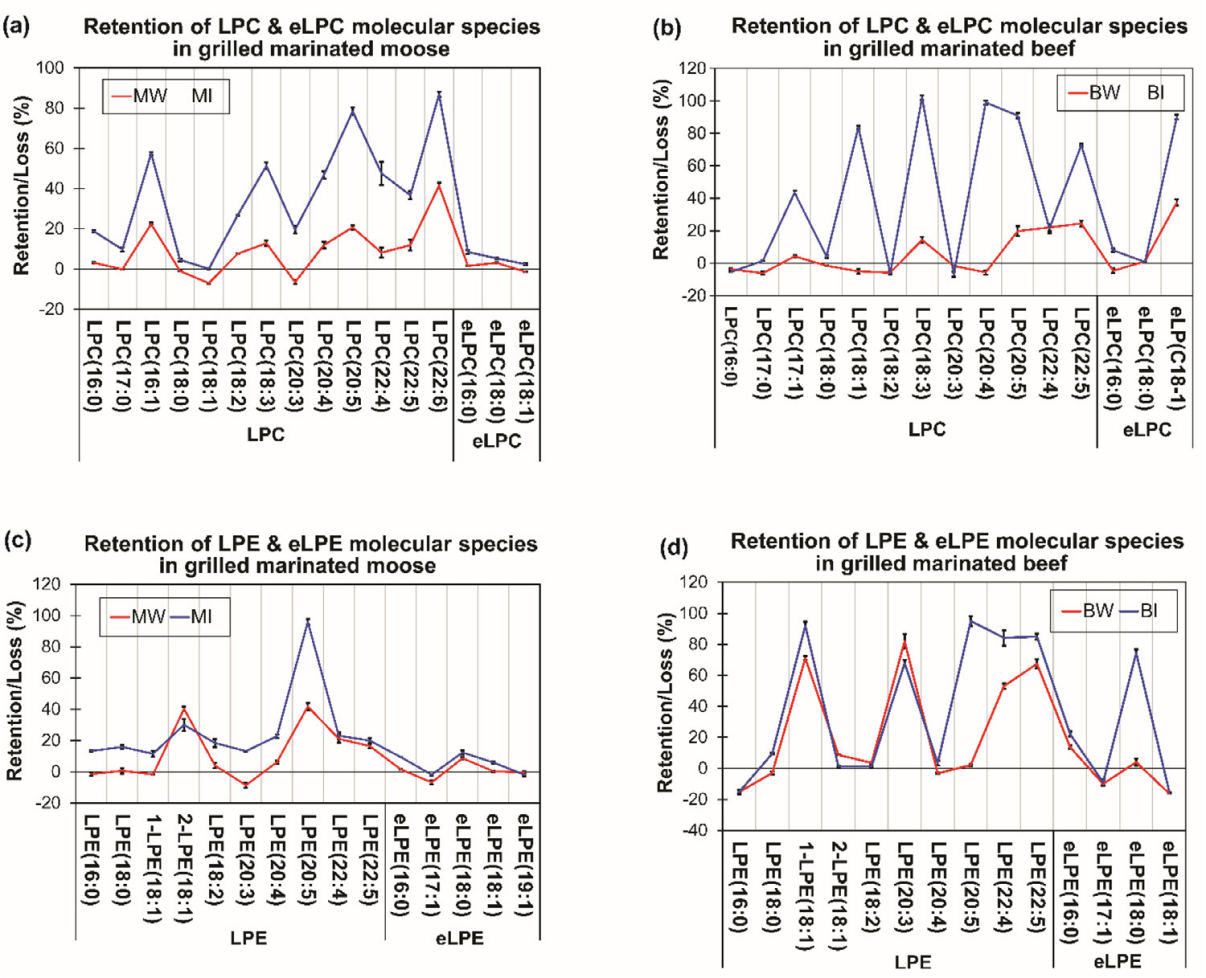
Parallel plots showing marination effects on lyso ether and ester PC and PE linked molecular species in grilled beef and moose meats. Values in plots represent means ± standard errors. Plots showing percentage changes (%) in LPC/eLPC **(a-b)** and LPE/eLPE **(c-d)** molecular species distribution in moose and beef respectively. [BC, MC] = unmarinated grilled beef and moose; [BI, MI] = Indian session ale-based marinated grilled beef and moose; [BW, MW] = Wheat ale-based marinated grilled beef and moose. PC = Phosphatidylcholine; PE = Phosphatidylethanolamine; *e* = Ether; *L* = Lyso.

**Table 1:** Pearson's correlation coefficients showing relationships between antioxidant activities, phenolic contents, volatile oxygenated terpenes, oxidation status, and the preserved ether PC (ePC) molecular species in grilled moose and beef meats.

ePC-Moose	LAA^a^	HAA^a^	LPC	HPC	LAA^b^	HAA^b^	LOS	HOS	1	2	3	4	5	6	7
16:0e/18:2	0.93^⁎⁎^	0.83^⁎⁎^	0.85^⁎⁎^	0.75*	0.95^⁎⁎^	0.96^⁎⁎^	−0.85^⁎⁎^	−0.80^⁎⁎^	0.89^⁎⁎^	0.15	0.89^⁎⁎^	0.92^⁎⁎^	0.81^⁎⁎^	0.96^⁎⁎^	0.82^⁎⁎^
16:0e/18:0	0.76*	0.08	0.83^⁎⁎^	0.96^⁎⁎^	0.43	0.34	−0.39	−0.19	0.54	0.32	0.49	0.40	0.64	0.70*	0.53
16:0e/19:1	0.85^⁎⁎^	0.29	0.91^⁎⁎^	0.98^⁎⁎^	0.53	0.50	−0.60	−0.42	0.63	0.21	0.56	0.51	0.69*	0.82^⁎⁎^	0.58
16:0e/20:4	0.79*	0.77*	0.73*	0.60	0.74*	0.84^⁎⁎^	−0.89^⁎⁎^	−0.92^⁎⁎^	0.72*	−0.16	0.67*	0.74*	0.58	0.86^⁎⁎^	0.58
16:0e/22:5	0.97^⁎⁎^	0.53	0.96^⁎⁎^	0.96^⁎⁎^	0.79*	0.76*	−0.75*	−0.59	0.83^⁎⁎^	0.21	0.79*	0.76*	0.80^⁎⁎^	0.94^⁎⁎^	0.75*
18:0e/20:3	0.89^⁎⁎^	0.27	0.88^⁎⁎^	0.99^⁎⁎^	0.60	0.53	−0.58	−0.35	0.66	0.31	0.62	0.55	0.69*	0.81^⁎⁎^	0.60
18:0e/22:5	0.93^⁎⁎^	0.61	0.93^⁎⁎^	0.83^⁎⁎^	0.90^⁎⁎^	0.85^⁎⁎^	−0.73*	−0.66	0.96^⁎⁎^	0.09	0.93^⁎⁎^	0.91^⁎⁎^	0.88^⁎⁎^	0.90^⁎⁎^	0.86^⁎⁎^
18:0e/18:0	0.63	−0.11	0.72*	0.90^⁎⁎^	0.26	0.16	−0.24	−0.02	0.40	0.33	0.35	0.23	0.52	0.54	0.40

ePC-Beef	LAA^a^	HAA^a^	LPC	HPC	LAA^b^	HAA^b^	LOS	HOS	1	2	3	4	5	6	7
16:0e/18:2	0.94^⁎⁎^	0.86^⁎⁎^	0.96^⁎⁎^	0.36	0.52	0.81^⁎⁎^	−0.77*	−0.81^⁎⁎^	0.92^⁎⁎^	0.99^⁎⁎^	0.89^⁎⁎^	0.95^⁎⁎^	0.98^⁎⁎^	0.98^⁎⁎^	0.80^⁎⁎^
16:0e/18:1	0.85^⁎⁎^	0.86^⁎⁎^	0.63	0.92^⁎⁎^	0.68*	0.90^⁎⁎^	−0.95^⁎⁎^	−0.83^⁎⁎^	0.75*	0.63	0.48	0.53	0.55	0.58	0.87^⁎⁎^
16:0e/18:0	0.85^⁎⁎^	0.90^⁎⁎^	0.65	0.88^⁎⁎^	0.68*	0.89^⁎⁎^	−0.84^⁎⁎^	−0.80^⁎⁎^	0.76*	0.63	0.52	0.57	0.56	0.58	0.74*
16:0e/19:1	0.91^⁎⁎^	0.92^⁎⁎^	0.75*	0.85^⁎⁎^	0.65	0.92^⁎⁎^	−0.96^⁎⁎^	−0.86^⁎⁎^	0.87^⁎⁎^	0.73*	0.59	0.63	0.66	0.69*	0.91^⁎⁎^
16:0e/20:4	0.36	0.26	0.62	−0.41	0.33	0.57	−0.00	−0.07	0.50	0.62	0.66	0.70*	0.67*	0.66	0.20
18:0e/18:2	0.17	0.18	0.38	−0.19	0.30	0.51	0.06	0.14	0.40	0.29	0.34	0.35	0.30	0.31	0.14
16:0e/22:5	0.77*	0.63	0.84^⁎⁎^	0.06	0.26	0.56	−0.53	−0.61	0.73*	0.93^⁎⁎^	0.87^⁎⁎^	0.95^⁎⁎^	0.95^⁎⁎^	0.94^⁎⁎^	0.60
18:0e/20:3	0.64	0.62	0.85^⁎⁎^	−0.11	0.57	0.30	−0.34	−0.56	0.75*	0.80^⁎⁎^	0.79*	0.78*	0.83^⁎⁎^	0.82^⁎⁎^	0.37
18:0e/22:5	0.71*	0.70*	0.93^⁎⁎^	0.04	0.6^7*^	0.26	−0.52	−0.58	0.89^⁎⁎^	0.88^⁎⁎^	0.84^⁎⁎^	0.85^⁎⁎^	0.90^⁎⁎^	0.90^⁎⁎^	0.63

Values with *: significant correlation (*P* < 0.05); ^⁎⁎^: significant correlation (*P* < 0.01). **1** = linalool; **2** = endo-borneol; **3** = terpinen-4-ol; **4** = terpineol; **5** = carvacrol; **6** = carvacrol isomer-1; **7** = carvacrol isomer-2. *P* = Phosphatidyl; *C* = Choline; LAA = Lipophilic antioxidant activity; HAA = Hydrophilic antioxidant activity; LPC = Lipophilic phenolic content; HPC = Hydrophilic phenolic content; LOS = Lipophilic oxidant status; HOS = Hydrophilic oxidant status *^a^ *= ABTS antioxidant activity. *^b^* = FRAP antioxidant activity; *e* = Ether.

**Table 2:** Pearson's correlation coefficients showing relationships between antioxidant activities, phenolic contents, volatile oxygenated terpenes, oxidation status, and the preserved diacyl PC (dPC) molecular species in grilled moose.

dPC-Species	LAA^a^	HAA^a^	LPC	HPC	LAA^b^	HAA^b^	LOS	HOS	1	2	3	4	5	6	7
16:1/16:1	0.68*	0.97^⁎⁎^	0.49	0.33	0.86^⁎⁎^	0.95^⁎⁎^	−0.82^⁎⁎^	−0.83^⁎⁎^	0.69*	0.06	0.72*	0.82^⁎⁎^	0.54	0.75*	0.61
16:0/16:1	0.98^⁎⁎^	0.68*	0.92^⁎⁎^	0.9^1**^	0.86^⁎⁎^	0.85^⁎⁎^	−0.80^⁎⁎^	−0.67*	0.83^⁎⁎^	0.260	0.82^⁎⁎^	0.81^⁎⁎^	0.81^⁎⁎^	0.98^⁎⁎^	0.78*
16:0/16:0	0.66	0.96^⁎⁎^	0.49	0.31	0.87^⁎⁎^	0.95^⁎⁎^	−0.79*	−0.83^⁎⁎^	0.70*	0.05	0.74*	0.83*	0.56	0.74*	0.63
17:1/16:0	0.95^⁎⁎^	0.65	0.96^⁎⁎^	0.89^⁎⁎^	0.85^⁎⁎^	0.84^⁎⁎^	−0.79*	−0.73*	0.89^⁎⁎^	0.10	0.85^⁎⁎^	0.84^⁎⁎^	0.85^⁎⁎^	0.97^⁎⁎^	0.82^⁎⁎^
16:1/18:3	0.92^⁎⁎^	0.81^⁎⁎^	0.86*	0.74*	0.92^⁎⁎^	0.95^⁎⁎^	−0.86^⁎⁎^	−0.85^⁎⁎^	0.89^⁎⁎^	0.05	0.88^⁎⁎^	0.91^⁎⁎^	0.80*	0.96^⁎⁎^	0.80^⁎⁎^
16:0/18:3	0.97^⁎⁎^	0.64	0.95^⁎⁎^	0.92^⁎⁎^	0.86^⁎⁎^	0.84^⁎⁎^	−0.79*	−0.67*	0.87^⁎⁎^	0.20	0.84^⁎⁎^	0.83^⁎⁎^	0.84^⁎⁎^	0.97^⁎⁎^	0.80^⁎⁎^
16:0/18:2	0.95^⁎⁎^	0.59	0.95^⁎⁎^	0.92^⁎⁎^	0.85^⁎⁎^	0.81^⁎⁎^	−0.73*	−0.59	0.88^⁎⁎^	0.24	0.85^⁎⁎^	0.83^⁎⁎^	0.86^⁎⁎^	0.94^⁎⁎^	0.82^⁎⁎^
18:0/16:0	0.70*	0.91^⁎⁎^	0.60	0.40	0.91^⁎⁎^	0.94^⁎⁎^	−0.77*	−0.73*	0.80^⁎⁎^	0.12	0.81^⁎⁎^	0.88^⁎⁎^	0.66	0.76*	0.73*
17:1/18:3	0.96*	0.59	0.97^⁎⁎^	0.94^⁎⁎^	0.81^⁎⁎^	0.79*	−0.77*	−0.65	0.85^⁎⁎^	0.18	0.80^⁎⁎^	0.79^⁎⁎^	0.82^⁎⁎^	0.96^⁎⁎^	0.78*
17:0/18:3	0.92*	0.82^⁎⁎^	0.85^⁎⁎^	0.73*	0.94^⁎⁎^	0.96^⁎⁎^	−0.87^⁎⁎^	−0.82^⁎⁎^	0.91^⁎⁎^	0.07	0.89^⁎⁎^	0.92^⁎⁎^	0.80*	0.95^⁎⁎^	0.81*
17:0/18:2	0.14	0.65	−0.19	−0.25	0.26	0.43	−0.54	−0.49	−0.02	−0.04	0.03	0.17	−0.23	0.19	−0.12
16:1/20:5	0.95^⁎⁎^	0.72*	0.90^⁎⁎^	0.83^⁎⁎^	0.84^⁎⁎^	0.88^⁎⁎^	−0.87^⁎⁎^	−0.82^⁎⁎^	0.84^⁎⁎^	0.051	0.80^⁎⁎^	0.82^⁎⁎^	0.76*	0.97^⁎⁎^	0.74*
18:3/18:3	0.96^⁎⁎^	0.72*	0.93^⁎⁎^	0.85^⁎⁎^	0.90^⁎⁎^	0.90^⁎⁎^	−0.83^⁎⁎^	−0.77*	0.91^⁎⁎^	0.101	0.88^⁎⁎^	0.89^⁎⁎^	0.83^⁎⁎^	0.97^⁎⁎^	0.82^⁎⁎^
16:0/20:5	0.94^⁎⁎^	0.45	0.96^⁎⁎^	0.99^⁎⁎^	0.72*	0.68*	−0.69*	−0.53	0.79*	0.23	0.74*	0.70*	0.79*	0.91^⁎⁎^	0.72*
18:3/18:2	0.81^⁎⁎^	0.13	0.89^⁎⁎^	0.97^⁎⁎^	0.53	0.43	−0.44	−0.36	0.66	0.25	0.61	0.52	0.73*	0.73*	0.63
16:0/20:4	0.80^⁎⁎^	0.16	0.93^⁎⁎^	0.96^⁎⁎^	0.55	0.46	−0.44	−0.36	0.72*	0.13	0.66	0.57	0.78*	0.75*	0.68*
18:1/18:3	0.62	−0.12	0.75^⁎⁎^	0.89^⁎⁎^	0.27	0.16	−0.22	−0.04	0.44	0.25	0.38	0.26	0.56	0.53	0.44
18:1/18:2	0.94^⁎⁎^	0.79*	0.86^⁎⁎^	0.76*	0.93^⁎⁎^	0.94^⁎⁎^	−0.87^⁎⁎^	−0.77*	0.90^⁎⁎^	0.12	0.88^⁎⁎^	0.91^⁎⁎^	0.79*	0.94^⁎⁎^	0.79*
18:3/20:5	0.95^⁎⁎^	0.47	0.93^⁎⁎^	0.96^⁎⁎^	0.78*	0.71*	−0.67*	−0.49	0.81^⁎⁎^	0.30	0.79*	0.74*	0.81^⁎⁎^	0.90^⁎⁎^	0.76*
18:2/20:5	0.84^⁎⁎^	0.19	0.89^⁎⁎^	0.99^⁎⁎^	0.53	0.45	−0.51	−0.33	0.63	0.25	0.58	0.50	0.69*	0.78*	0.59
16:0/22:6	0.97^⁎⁎^	0.69*	0.93^⁎⁎^	0.87^⁎⁎^	0.89^⁎⁎^	0.88^⁎⁎^	−0.84^⁎⁎^	−0.74*	0.90^⁎⁎^	0.13	0.87^⁎⁎^	0.87^⁎⁎^	0.82*	0.97^⁎⁎^	0.80^⁎⁎^
18:2/20:4	0.74*	0.03	0.82^⁎⁎^	0.95^⁎⁎^	0.39	0.30	−0.39	−0.16	0.52	0.27	0.46	0.36	0.60	0.66	0.49
16:0/22:5	0.28	0.76*	0.17	−0.09	0.59	0.68*	−0.57	−0.55	0.48	−0.12	0.45	0.59	0.26	0.34	0.36
18:0/20:4	0.88^⁎⁎^	0.78*	0.76*	0.62	0.89^⁎⁎^	0.92^⁎⁎^	−0.91^⁎⁎^	−0.78*	0.87^⁎⁎^	−0.03	0.83^⁎⁎^	0.87^⁎⁎^	0.66	0.84^⁎⁎^	0.68*
18:3/22:5	0.78*	0.08	0.85^⁎⁎^	0.96^⁎⁎^	0.41	0.35	−0.47	−0.29	0.56	0.15	0.48	0.40	0.60	0.70*	0.49
20:4/20:4	0.64	−0.13	0.72*	0.90^⁎⁎^	0.25	0.15	−0.26	−0.05	0.4	0.25	0.34	0.23	0.50	0.54	0.38
22:5/18:2	0.71*	−0.02	0.82^⁎⁎^	0.93^⁎⁎^	0.34	0.26	−0.40	−0.17	0.52	0.15	0.43	0.34	0.55	0.61	0.44

Values with *: significant correlation (*P* < 0.05); **: significant correlation (*P* < 0.01). **1** = linalool; **2** = endo-borneol; **3** = terpinen-4-ol; **4** = terpineol; **5** = carvacrol; **6** = carvacrol isomer-1; **7** = carvacrol isomer-2. *P* = Phosphatidyl; *C* = Choline; LAA = Lipophilic antioxidant activity; HAA = Hydrophilic antioxidant activity; LPC = Lipophilic phenolic content; HPC = Hydrophilic phenolic content; LOS = Lipophilic oxidant status; HOS = Hydrophilic oxidant status

a = ABTS antioxidant activity.

b = FRAP antioxidant activity; *d* = Diacyl.

**Table 3:** Pearson's correlation coefficients showing relationships between antioxidant activities, phenolic contents, volatile oxygenated terpenes, oxidation status, and the preserved diacyl PC (dPC) molecular species in grilled beef.

dPC-Species	LAA^a^	HAA^a^	LPC	HPC	LAA^b^	HAA^b^	LOS	HOS	1	2	3	4	5	6	7
16:0/16:0	0.69*	0.72*	0.41	0.99^⁎⁎^	0.57	0.76*	−0.86^⁎⁎^	−0.66	0.58	0.41	0.26	0.31	0.31	0.35	0.78*
17:1/16:0	−0.01	0.06	−0.34	0.76*	0.19	0.16	−0.32	−0.05	−0.14	−0.33	−0.41	−0.39	−0.42	−0.39	0.22
16:1/18:3	0.39	0.32	0.69*	−0.44	0.00	0.18	−0.08	−0.26	0.55	0.66	0.69*	0.68*	0.72*	0.71*	0.21
16:0/18:1	0.43	0.52	0.12^⁎⁎^	0.97^⁎⁎^	0.55	0.62	−0.62*	−0.52	0.30	0.09	−0.03	−0.01	−0.00	0.03	0.53
18:0/16:0	0.89^⁎⁎^	0.94^⁎⁎^	0.76*	0.72*	0.73*	0.93^⁎⁎^	−0.79*	−0.88^⁎⁎^	0.80^⁎⁎^	0.73*	0.64	0.67	0.69*	0.70*	0.66
17:0/18:3	−0.36	−0.40	0.01	−0.92^⁎⁎^	−0.47	−0.49	0.60	0.42	−0.15	−0.04	0.08	0.03	0.05	0.03	−0.45
17:0/18:1	0.50	0.56	0.22	0.96^⁎⁎^	0.54	0.66	−0.77*	−0.57	0.40	0.19	0.06	0.07	0.10	0.14	0.66
16:1/20:5	0.67*	0.61	0.89^⁎⁎^	−0.12	0.23	0.49	−0.38	−0.53	0.79*	0.85^⁎⁎^	0.84^⁎⁎^	0.85^⁎⁎^	0.89^⁎⁎^	0.88^⁎⁎^	0.47
18:1/18:1	0.62	0.71*	0.34	0.98^⁎⁎^	0.66	0.78*	−0.77*	−0.68*	0.49	0.30	0.19	0.21	0.22	0.25	0.61
18:0/18:1	0.56	0.60	0.26	0.99^⁎⁎^	0.51	0.66	−0.79*	−0.56	0.44	0.25	0.12	0.16	0.16	0.20	0.70*
18:3/20:5	0.22	0.05	0.33	−0.42	0.01	0.09	−0.03	−0.20	0.14	0.48	0.47	0.52	0.54	0.50	0.07
18:1/19:0	0.60	0.66*	0.29	1.00^⁎⁎^	0.60	0.73*	−0.79*	−0.63	0.46	0.29	0.16	0.20	0.20	0.23	0.66
18:0/20:4	0.55	0.62	0.23	0.99^⁎⁎^	0.68*	0.62	−0.74*	−0.61	0.38	0.23	0.10	0.14	0.14	0.17	0.58
16:0/22:6	0.42	0.24	0.58	−0.36	0.23	0.02	−0.14	−0.24	0.41	0.70*	0.69*	0.76*	0.75*	0.72*	0.26

Values with *: significant correlation (*P* < 0.05); **: significant correlation (*P* < 0.01). **1** = linalool; **2** = endo-borneol; **3** = terpinen-4-ol; **4** = terpineol; **5** = carvacrol; **6** = carvacrol isomer-1; **7** = carvacrol isomer-2. *P* = Phosphatidyl; *C* = Choline; LAA = Lipophilic antioxidant activity; HAA = Hydrophilic antioxidant activity; LPC = Lipophilic phenolic content; HPC = Hydrophilic phenolic content; LOS = Lipophilic oxidant status; HOS = Hydrophilic oxidant status.

a = ABTS antioxidant activity.

b = FRAP antioxidant activity; *d* = Diacyl.

**Table 4:** Pearson's correlation coefficients showing relationships between antioxidant activities, phenolic contents, volatile oxygenated terpenes, oxidation status, and the preserved ether and diacyl PE molecular species in grilled moose.

dPE/ePE	LAA^a^	HAA^a^	LPC	HPC	LAA^b^	HAA^b^	LOS	HOS	1	2	3	4	5	6	7
16:1/20:5	0.95⁎⁎	0.80⁎⁎	0.87⁎⁎	0.79*	0.92⁎⁎	0.94⁎⁎	−0.87⁎⁎	−0.80⁎⁎	0.87⁎⁎	0.14	0.86⁎⁎	0.88⁎⁎	0.79⁎⁎	0.98⁎⁎	0.79*
16:1/20:4	0.79*	0.89⁎⁎	0.63	0.49	0.97⁎⁎	0.98⁎⁎	−0.77*	−0.76*	0.84⁎⁎	0.14	0.88⁎⁎	0.93⁎⁎	0.72*	0.82⁎⁎	0.77*
18:2/18:3	0.84⁎⁎	0.25	0.91⁎⁎	0.98⁎⁎	0.52	0.49	−0.57	−0.47	0.63	0.12	0.57	0.51	0.67*	0.81⁎⁎	0.58
18:2/18:2	0.96⁎⁎	0.68*	0.92⁎⁎	0.85⁎⁎	0.88⁎⁎	0.88⁎⁎	−0.80⁎⁎	−0.79*	0.88⁎⁎	0.07	0.86⁎⁎	0.86*	0.82⁎⁎	0.97⁎⁎	0.80⁎⁎
18:1/18:2	0.80⁎⁎	0.93⁎⁎	0.68*	0.51	0.93⁎⁎	0.99⁎⁎	−0.86⁎⁎	−0.86⁎⁎	0.83⁎⁎	0.05	0.84⁎⁎	0.91⁎⁎	0.69*	0.86⁎⁎	0.74*
18:0/18:2	0.67*	0.94⁎⁎	0.54	0.33	0.88⁎⁎	0.96⁎⁎	−0.80⁎⁎	−0.86⁎⁎	0.76*	−0.03	0.78*	0.87⁎⁎	0.60	0.75*	0.67*
16:1/18:2	0.80⁎⁎	0.92⁎⁎	0.69*	0.52	0.93⁎⁎	0.99⁎⁎	−0.8^5^⁎⁎	−0.88⁎⁎	0.84⁎⁎	0.01	0.84⁎⁎	0.91⁎⁎	0.70*	0.86⁎⁎	0.74*
18:3/20:4	0.88⁎⁎	0.33	0.84⁎⁎	0.98⁎⁎	0.57	0.53	−0.61	−0.35	0.59	0.39	0.56	0.50	0.64	0.83⁎⁎	0.56
18:1/20:5	0.97⁎⁎	0.60	096⁎⁎	0.94⁎⁎	0.82⁎⁎	0.81⁎⁎	−0.78*	−068*	0.85⁎⁎	0.17	0.81⁎⁎	0.79*	0.82⁎⁎	0.97⁎⁎	0.78*
18:0/20:5	0.96⁎⁎	0.53	0.96⁎⁎	0.97⁎⁎	0.77*	0.74*	−0.73*	−0.60	0.81⁎⁎	0.22	0.77*	0.74*	0.81⁎⁎	0.95⁎⁎	0.75*
18:0/20:4	0.86⁎⁎	0.26	0.88⁎⁎	0.99⁎⁎	0.58	0.51	−0.54	−0.33	0.64	0.34	0.60	0.53*	0.71*	0.81⁎⁎	0.62
18:0/20:3	0.78*	0.11	0.85⁎⁎	0.97⁎⁎	0.44	0.37	−0.43	−0.27	0.56	0.26	0.51	0.42	0.64	0.72*	0.53
18:0/20:2	0.93⁎⁎	0.82⁎⁎	0.85⁎⁎	0.76*	0.95⁎⁎	0.96⁎⁎	−0.85⁎⁎	−0.78*	0.90⁎⁎	0.15	0.90⁎⁎	0.92⁎⁎	0.82⁎⁎	0.96⁎⁎	0.83⁎⁎
18:0/22:5	0.75*	0.93⁎⁎	0.64	0.45	0.91⁎⁎	0.97⁎⁎	−0.84⁎⁎	−0.88⁎⁎	0.80⁎⁎	−0.02	0.81⁎⁎	0.89⁎⁎	0.65	0.82⁎⁎	0.71*
16:1/18:1	0.18	0.79*	0.01	−0.24	0.55	0.66	−0.49	−0.64	0.36	−0.17	0.41	0.54	0.17	0.28	0.29
16:0/18:1	0.64	0.93⁎⁎	0.54	0.31	0.88⁎⁎	0.94⁎⁎	−0.77*	−0.83⁎⁎	0.77*	−0.02	0.79*	0.87⁎⁎	0.61	0.72*	0.68*
18:1/20:0	0.90**	0.37	0.94⁎⁎	0.97⁎⁎	0.66	0.63	−0.63	−0.58	0.76*	0.09	0.71*	0.66	0.77*	0.88⁎⁎	0.69*
18:0/16:0	0.98⁎⁎	0.67*	0.94⁎⁎	0.90⁎⁎	0.87⁎⁎	0.56⁎⁎	−0.81⁎⁎	−0.71*	0.87⁎⁎	0.17	0.84⁎⁎	0.84⁎⁎	0.83⁎⁎	0.98⁎⁎	0.80⁎⁎
17:0/18:1	0.67*	0.96⁎⁎	0.52	0.34	0.84⁎⁎	0.94⁎⁎	−0.84⁎⁎	−0.83⁎⁎	0.69*	0.03	0.70*	0.8⁎⁎	0.532	0.76*	0.60
18:0/18:0	0.95⁎⁎	0.52	0.97⁎⁎	0.96⁎⁎	0.76*	0.74*	−0.74*	−0.66	0.82*	0.12	0.77*	0.75*	0.81⁎⁎	0.94⁎⁎	0.75*
18:0/20:0	0.18	−0.54	0.31	0.55	−0.16	−0.32	0.25	0.45	−0.01	0.339	−0.04	−0.18	0.18	0.06	0.06

Values with *: significant correlation (*P* < 0.05).

⁎⁎ : significant correlation (*P* < 0.01). **1** = linalool; **2** = endo-borneol; **3** = terpinen-4-ol; **4** = terpineol; **5** = carvacrol; **6** = carvacrol isomer-1; **7** = carvacrol isomer-2. *P* = Phosphatidyl; *E* = Ethanolamine; LAA = Lipophilic antioxidant activity; HAA = Hydrophilic antioxidant activity; LPC = Lipophilic phenolic content; HPC = Hydrophilic phenolic content; LOS = Lipophilic oxidant status; HOS = Hydrophilic oxidant status.

a = ABTS antioxidant activity.

b = FRAP antioxidant activity; *d* = Diacyl; *e* = Ether.

**Table 5:** Pearson's correlation coefficients showing relationships between antioxidant activities, phenolic contents, volatile oxygenated terpenes, oxidation status, and the preserved ether and diacyl PE molecular species in grilled beef.

dPE/ePE	LAA^a^	HAA^a^	LPC	HPC	LAA^b^	HAA^b^	LOS	HOS	1	2	3	4	5	6	7
16:0/18:2	0.86^⁎⁎^	0.86^⁎⁎^	0.75*	0.82^⁎⁎^	0.73*	0.93^⁎⁎^	−0.92^⁎⁎^	−0.83^⁎⁎^	0.92^⁎⁎^	0.97^⁎⁎^	0.85^⁎⁎^	0.92^⁎⁎^	0.94^⁎⁎^	0.95^⁎⁎^	0.86^⁎⁎^
16:1/20:5	0.74*	0.70*	0.93^⁎⁎^	0.02	0.34	0.59	−0.49	−0.62	0.51	0.32	0.18	0.23	0.23	0.26	0.74*
16:1/20:4	0.85^⁎⁎^	0.83^⁎⁎^	0.95^⁎⁎^	0.24	0.43	0.71*	−0.622	−0.69*	0.67*	0.50	0.37	0.43	0.41	0.44	0.80^⁎⁎^
18:2/18:3	0.07	0.02	0.37	−0.70*	−0.08	−0.28	0.28	0.02	−0.26	−0.49	−0.55	−0.57	−0.57	−0.54	0.07
16:0/20:4	0.90^⁎⁎^	0.92^⁎⁎^	0.81^⁎⁎^	0.78*	0.68*	0.93^⁎⁎^	−0.93^⁎⁎^	−0.89^⁎⁎^	0.9^7**^	0.91^⁎⁎^	0.78*	0.83^⁎⁎^	0.87^⁎⁎^	0.89^⁎⁎^	0.93^⁎⁎^
18:2/18:2	0.00	−0.00	−0.13	0.51	0.45	0.26	−0.25	−0.06	0.19	0.54	0.55	0.64	0.60	0.56	0.08
18:1/18:2	0.67*	0.64	0.85^⁎⁎^	−0.08	0.19	0.47	−0.39	−0.55	0.42	0.17	0.05	0.07	0.07	0.11	0.64
18:0/18:2	−0.01	−0.11	0.20	−0.72*	−0.19	−0.50	0.39	0.19	−0.43	−0.63	−0.64	−0.70*	−0.68*	−0.66	−0.27
18:3/20:4	−0.13	−0.21	0.20	−0.85^⁎⁎^	−0.29	−0.36	0.48	0.27	−0.51	−0.63	−0.66	−0.64	−0.69*	−0.68*	−0.20
18:1/20:5	0.74*	0.69*	0.92^⁎⁎^	−0.01	0.29	0.56	−0.46	−0.60	0.48	0.28	0.14	0.19	0.18	0.22	0.69*
18:0/20:5	0.79*	0.86^⁎⁎^	0.71*	0.77*	0.66	0.87^⁎⁎^	−0.90^⁎⁎^	−0.86^⁎⁎^	0.96^⁎⁎^	0.87^⁎⁎^	0.75*	0.78*	0.83^⁎⁎^	0.85^⁎⁎^	0.95^⁎⁎^
18:0/20:4	0.76*	0.80^⁎⁎^	0.52	0.97^⁎⁎^	0.63	0.83^⁎⁎^	−0.89^⁎⁎^	−0.74*	0.95^⁎⁎^	0.95^⁎⁎^	0.88^⁎⁎^	0.91^⁎⁎^	0.95^⁎⁎^	0.95^⁎⁎^	0.71*
18:0/20:3	0.79*	0.81^⁎⁎^	0.55	0.96^⁎⁎^	0.62	0.84^⁎⁎^	−0.91^⁎⁎^	−0.77*	0.94^⁎⁎^	0.94^⁎⁎^	0.87^⁎⁎^	0.89^⁎⁎^	0.94^⁎⁎^	0.95^⁎⁎^	0.71*
18:0/20:2	0.76*	0.74*	0.49	0.69*	0.22	0.58	−0.64	−0.51	0.69*	0.58	0.57	0.55	0.57	0.58	0.33
16:1/18:1	0.87^⁎⁎^	0.86*	0.96^⁎⁎^	0.29	0.82^⁎⁎^	0.43	−0.67*	−0.75*	0.72*	0.52	0.39	0.43	0.44	0.47	0.84^⁎⁎^
16:1/16:1	0.38	0.44	0.05	0.96^⁎⁎^	0.45	0.53	−0.64	−0.42	0.68*	0.7^8*^	0.78*	0.79*	0.82^⁎⁎^	0.81^⁎⁎^	0.34
16:0/16:1	0.51	0.56	0.20	0.97^⁎⁎^	0.54	0.64	−0.72*	−0.49	0.76*	0.87^⁎⁎^	0.85^⁎⁎^	0.89^⁎⁎^	0.90^⁎⁎^	0.89^⁎⁎^	0.43
16:0/18:1	0.60	0.65	0.31	1.00^⁎⁎^	0.60	0.73*	−0.81^⁎⁎^	−0.61	0.86^⁎⁎^	0.92^⁎⁎^	0.88^⁎⁎^	0.91^⁎⁎^	0.94^⁎⁎^	0.93^⁎⁎^	0.58
18:1/20:0	0.79*	0.73*	0.92^⁎⁎^	0.06	0.24	0.58	−0.51	−0.64	0.51	0.29	0.17	0.18	0.21	0.24	0.66
16:0/18:0	0.94^⁎⁎^	0.94^⁎⁎^	0.81^⁎⁎^	0.81^⁎⁎^	0.65	0.93^⁎⁎^	−0.94^⁎⁎^	−0.87^⁎⁎^	0.97^⁎⁎^	0.92^⁎⁎^	0.81^⁎⁎^	0.85^⁎⁎^	0.88^⁎⁎^	0.90^⁎⁎^	0.87^⁎⁎^
17:0/18:1	0.31	0.37	−0.02	0.93^⁎⁎^	0.46	0.49	−0.61	−0.39	0.64	0.76^⁎⁎^	0.77*	0.77*	0.81^⁎⁎^	0.80^⁎⁎^	0.31
18:0/18:0	0.53	0.36	0.52	0.12	0.17	0.37	−0.27	−0.22	0.15	0.29	0.25	0.29	0.27	0.26	0.08
18:0/20:0	0.35	0.30	0.39	0.37	0.62	0.53	−0.43	−0.36	0.35	0.63	0.55	0.67*	0.63	0.61	0.47
18:0e/22:5	0.59	0.52	0.79*	−0.25	0.03	0.32	−0.22	−0.39	0.23	−0.01	−0.11	−0.10	−0.10	−0.06	0.42

Values with *: significant correlation (*P* < 0.05); **: significant correlation (*P* < 0.01). **1** = linalool; **2** = endo-borneol; **3** = terpinen-4-ol; **4** = terpineol; **5** = carvacrol; **6** = carvacrol isomer-1; **7** = carvacrol isomer-2. *P* = Phosphatidyl; *E* = Ethanolamine; LAA = Lipophilic antioxidant activity; HAA = Hydrophilic antioxidant activity; LPC = Lipophilic phenolic content; HPC = Hydrophilic phenolic content; LOS = Lipophilic oxidant status; HOS = Hydrophilic oxidant status.

a = ABTS antioxidant activity.

b = FRAP antioxidant activity; *d* = Diacyl; *e* = Ether.

## 2 Experimental Design, Materials, and Methods

### 2.1 Standards and reagents

SPLASH® Lipidomix® Mass Spec Standard (Product Number 330707) was purchased from Avanti Polar Lipids (Alabama, USA), and was used to generate standard calibration curves for quantification of PC and PE in grilled meats. All other reagents were purchased from Sigma Aldrich (Ontario, Canada) and were of analytical grade. All solvents used were of HPLC grade from VWR International (Ontario, Canada).

### 2.2 Sample preparation of marinades

Detailed procedures for marinade composition and grilling conditions are same as described in our previous publication [1,3]. Briefly, herbs, spices, India ale and Wheat ale beers were purchased from a supermarket in Corner Brook, Newfoundland and Labrador, Canada. India ale contained 4.3% alcohol, water, malted barley, and hops; Wheat ale contained 5.2% alcohol, water, malted wheat, barley, orange, lemon, lime peel, coriander, Cascade and Willamette hops. Each marinade contained 341 mL beer, 1 g oregano, 1 g parsley, 4 g mustard, 2 g salt, 8 g pepper, 1 g garlic, 25 mL olive oil, 15 mL vinegar and 25 g fresh onions. “Beef (B) and moose (M) striploin steaks (*Longissimus thoracis et lumborum*) were obtained from a local market and from Newfoundland and Labrador Department of Natural Resources, respectively. Moose steaks from 4 different animals and 4 different beef steak batches were used to mitigate any inherent variability of the meat sources. Steaks (1 lb) of beef and moose meat from different batches were cut and divided into four replicates (*n* = 4) per treatment. The steaks were divided into three groups as follows: control group contained unmarinated samples, (C), treatment group contained samples marinated with either India ale-based marinade (I) or Wheat ale beer-based marinade (W). Marination was performed by adding 600 mL of each beer-based marinade to beef and moose steaks for 12 h at 4 °C in zip lock closed plastic bags” [3]. Meat samples were grilled at 200–250 °C for 25 min reaching an internal temperature of 75 °C. The grill was thoroughly cleaned between samples to avoid any possible contamination of marinade flavors. Ethics approval [20160041] was issued by Memorial University Animal Care Committee. All experiments conformed to relevant guidelines and regulations [1].

### 2.3 Extraction and analysis of antioxidants, polyphenols and pro-oxidants

Detailed procedures for extraction and colorimetric analysis of antioxidants, polyphenols and pro-oxidants are same as described in our previous publications [3,5]. Briefly, total polyphenol and antioxidant analyses were based on the Folin-Ciocalteu (FC) and ABTS antioxidant methods respectively [6,7], while oxidation status was assessed by the method of Erel, 2005 [8]. Results from the ABTS method were corroborated by measuring total antioxidant content using Ferric reducing antioxidant power (FRAP) method [9]. Four experimental replicates of meat treatments were used for colorimetric analyses (*n* = 4)[1, 3].

### 2.4 Extraction and LC-MS analysis of meat lipids

Prior to lipid extraction, grilled meat samples were homogenized, and lipids extracted in triplicate (*n* = 3) from ground portions (1 g) according to the Folch method by mixing with 2 mL chloroform/methanol (2:1, v/v) [2,3]. To this mixture, 1 mL of 0.25% KCl was added and the sample vortexed and centrifuged. The organic phase was then recovered and pooled into clean pre-weighed vials, and the extracts evaporated to dryness under nitrogen to determine the extracted lipid weight. Prior to LC–MS analysis, meat lipids were reconstituted in 1 mL of chloroform/methanol (2:1). Procedures for LC–MS analysis are same as described in our previous publications [10,11]. Lipid concentrations were expressed on mg/100 g meat and nmol% basis. The difference between lipid molecular species levels in marinated and unmarinated grilled meat samples was calculated using the equation [1]:

$$\text{\%}\;\mathrm{retention/loss}=\left(\frac{marinated-unmarinated}{unmarinated}\right)*100$$

### 2.5 Extraction and analysis of meat volatile oxygenated terpene components by SPME-GC/MS

The procedure for extraction and analysis of meat volatile components including oxygenated terpenes by solid phase microextraction coupled to gas chromatography/mass spectrometry (SPME-GC/MS) is the same as described in our previous publications [3,5]. Briefly, three experimental replicates of meat treatments were used for SPME-GC/MS analysis of volatile oxygenated terpenes (*n* = 3). Detailed results and discussion of SPME-GC/MS analysis of volatile oxygenated terpenes in grilled meat samples are provided in our previous publication [1,5].

### 2.6 Data processing

Processing of raw lipid data using LipidSearch 4.2 (Mitsui Knowledge Industry, Tokyo, Japan) and Xcalibur 4.0 (Thermo Fisher Scientific, Ontario, Canada) softwares are same as described in our previous publications [1,10,11]. Briefly, LipidSearch parameters used for processing were as follows: target database: Q Exactive; precursor tolerance: 5 ppm; product tolerance: 5 ppm; product ion threshold: 5%; m-score threshold: 2; Quan *m/z* tolerance: ±5 ppm; Quan RT (retention time) range: ± 1 min; use of all isomer filter and ID quality filters A, B, and C; Adduct ions: +NH_4_ and +*H* for positive ion mode, +HCOO and −*H*, for negative ion mode. The alignment parameters were first optimized using lipid standards before being applied to targeted lipidomics analysis. Positions of the fatty acyls (fatty acids) present in the GPL molecular species found in the samples evaluated were identified based on the fragmentation patterns of the MS/MS spectra, and manually confirmed using Xcalibur 4.0 according to the well-recognized rules established by tandem mass spectrometry [12].

## Declaration of Competing Interest

The authors declare that they have no known competing financial interests or personal relationships which have, or could be perceived to have, influenced the work reported in this article.

## Appendix Supplementary Materials

Image, application 1
